# C3 mutations and poor pegcetacoplan response in paroxysmal nocturnal hemoglobinuria

**DOI:** 10.3389/fimmu.2025.1723596

**Published:** 2025-11-26

**Authors:** Santiago Rodríguez de Córdoba, Andrea Reparaz Suevos, Silvia González Sanz, Francisco J. Fernández, M. Cristina Vega, Enrique Colado Varela, Alejandro Lada Colunga, Juan Carlos Vallejo Llamas, Ana Pilar Gonzalez-Rodríguez

**Affiliations:** 1 Centro de Investigaciones Biológicas Margarita Salas (CIB-MS), Madrid, Spain; 2 Hospital Universitario Central de Asturias, Universidad de Oviedo, Oviedo, Spain; 3 Hospital Universitario de Salamanca, Salamanca, Spain

**Keywords:** complement, genetics, pegcetacoplan, eculizumab, AP50

## Abstract

**Introduction:**

Paroxysmal nocturnal hemoglobinuria (PNH) is treated with complement inhibitors, yet incomplete responses remain a challenge. Terminal inhibition with eculizumab prevents intravascular hemolysis but often leaves residual extravascular hemolysis, while proximal inhibitors such as pegcetacoplan mitigate extravascular hemolysis though intravascular hemolysis may persist. Understanding resistance mechanisms is critical to guide therapy.

**Methods:**

We report a PNH patient with incomplete responses to both eculizumab and pegcetacoplan. Genetic analysis revealed a mutation in the C3 MG-ring. Functional assays assessed the effects of this variant on C3b inactivation by complement regulators and on pegcetacoplan binding. Additional MG-ring variants were analyzed to explore broader relevance.

**Results:**

The C3 MG-ring mutation rendered C3b resistant to inactivation and reduced pegcetacoplan binding, explaining persistent hemolysis under both therapeutic approaches. Other MG-ring variants produced similar effects, indicating that this structural domain represents a recurrent vulnerability of C3 and a potential mechanism of resistance to complement inhibition.

**Discussion:**

These findings show that C3 MG-ring mutations can undermine both terminal and proximal complement inhibitors, directly impacting treatment efficacy in PNH. Complement genetic testing, combined with functional readouts, may help identify patients at risk of suboptimal responses and support personalized therapeutic strategies.

## Introduction

Paroxysmal nocturnal hemoglobinuria (PNH) is a rare clonal hematopoietic disorder characterized by complement-mediated intravascular hemolysis, bone marrow failure, and high thrombotic risk. It arises from somatic *PIGA* mutations causing loss of glycosylphosphatidylinositol (GPI)-anchored proteins, including the complement regulators CD55 and CD59. Without these protective complement regulators, PNH erythrocytes (E-PNH) become highly susceptible to complement-mediated lysis (1, 2).

Eculizumab, a monoclonal antibody against complement C5, transformed PNH treatment by blocking terminal complement activation and intravascular hemolysis, improving hemoglobin (Hb), and reducing transfusion needs and thrombosis (3, 4). However, in addition to a lack of response due to a rare C5 polymorphism in Asian populations (5), many patients show suboptimal responses with fatigue, anemia, and recurrent intravascular hemolysis. This results from massive C3 opsonization of E-PNH under complement activation, driving extravascular clearance via opsonophagocytosis (6, 7) and eventually bypassing eculizumab to cause intravascular hemolysis (8). Such opsonization and poor responses are exacerbated by low erythrocyte complement receptor 1 (CR1) expression (9, 10) and may also involve rare factor H (FH) variants (11).

Proximal complement inhibitors, such as the C3 inhibitor pegcetacoplan and the factor B (FB) inhibitor iptacopan, provide broader blockade and superior control of intra- and extravascular hemolysis compared to terminal inhibitors (12–15). However, breakthrough intravascular hemolysis still occurs on pegcetacoplan, particularly during infections or inflammation; some patients respond to higher doses, but others remain with hemolytic crises (16, 17).

The implementation of anti-complement therapies has been an extraordinary advance in PNH, setting a new standard of care. Nevertheless, a proportion of patients display incomplete responses that remain poorly understood, particularly under proximal C3 blockade with pegcetacoplan. At present, no systematic molecular, genetic, or functional complement investigations are performed to clarify the mechanisms of these suboptimal responses. Filling this critical gap is an urgent and unmet need to guide precision use of anti-complement agents and to tailor treatment strategies to individual patients.

Here we report a PNH patient with incomplete responses to eculizumab and pegcetacoplan, carrying a heterozygous C3 gain-of-function (GOF) mutation (c.1514G>A; p.Arg505His) that reduces pegcetacoplan binding and impairs C3b inactivation by FH, thereby dysregulating the alternative pathway and increasing E-PNH opsonization. These findings provide a mechanistic explanation for the persistent hemolytic activity observed in our patient under both terminal and proximal inhibition, and they prompted us to demonstrate that other C3 GOF mutations also exhibit impaired binding to pegcetacoplan, suggesting that this resistance mechanism may have a broader clinical impact.

## Methods

### Patient case

Our patient, diagnosed in 2004 with severe aplastic anemia with a small PNH clone (1%), responded to anti-thymocyte globulin (ATG) and cyclosporine (CSA). CSA was discontinued in 2007, but reintroduction was necessary in 2010 due to cytopenia and mild marrow aplasia; PNH clone expansion (6%) was also documented. By 2017, the clone had expanded significantly, confirming PNH disease.

Eculizumab was started in 2019 for high hemolytic activity but provided only partial benefit, with persistent fatigue despite hemoglobin improvement and lactate dehydrogenase (LDH) normalization. The patient transitioned to crovalimab in 2021 in the context of a clinical trial, but by March 2022, extravascular hemolysis (Coombs C3+, hemoglobin (Hb) 10.3 g/dL, indirect bilirubin 8 mg/dL, normal LDH) was documented; PNH clone was 87%. Simultaneously, CSA had to be restarted for cytopenia secondary to bone marrow hypoplasia.

Pegcetacoplan was initiated in January 2023, achieving an excellent hematologic response (Hb 14.3 g/dL, indirect bilirubin 1.1 mg/dL, PNH clone >95%). In September 2023, CSA was discontinued, and recurrent hemolytic crises developed, often infection-triggered (COVID-19, Influenza, respiratory infections) and occasionally spontaneous. These episodes (Hb nadirs 7.3–11.6 g/dL, LDH up to 1400 U/L) were consistently controlled by increasing pegcetacoplan dosing from twice to three times weekly. In more severe flares, including a December 2024 crisis (Hb 8.6 g/dL, LDH 1161 U/L), intensification to three consecutive daily pegcetacoplan doses achieved resolution.

Again, due to evidence of central cytopenia, CSA was reintroduced in January 2025. Recurrent episodes continued to be managed successfully with short-term pegcetacoplan intensifications. Despite increased pegcetacoplan dosing, our patient continues to show incomplete inhibition, remaining at risk for new crises during strong complement activation. Switching to iptacopan, another proximal inhibitor that has shown benefits in some patients (18), was initiated in September 2025, resulting in complete inhibition of the alternative pathway, full normalization of Hb and LDH levels and disappearance of the clinical symptoms associated with intravascular hemolysis.

### Complement studies

Plasma levels of complement proteins C3, C4, factor H (FH), factor I (FI), factor B (FB) and Properdin, presence of biomarkers of complement activation iC3b, C3dg and sC5b-9, and the alternative pathway 50% hemolytic complement activity (AP50) were measured in serum or plasma samples of the patient using routine assays performed in the Complement Diagnostic Laboratory at the Centro de Investigaciones Biologicas Margarita Salas (CIB-CSIC).

### Complement genetics analyses

The patient was analyzed for genetic variants using an in-house next-generation sequencing (NGS) panel that includes 50 genes: *C1QA*, *C1QB*, *C1QC*, *C1R*, *C1S*, *C2*, *C3*, *C4A*, *C4BPA*, *C4BPB*, *C5*, *C6*, *C7*, *C8A, C8B*, *C8G*, *C9*, *CD46*, *CD55*, *CD59*, *CFB*, *CFD*, *CFH*, *CFHR1*, *CFHR2*, *CFHR3*, *CFHR4*, *CFHR5*, *CFI*, *CFP*, *CLU*, *CR1*, *CR2*, *FCN1*, *FCN2*, *FCN3*, *ITGAX*, *ITGAM*, *ITGB2*, *MASP1*, *MASP2*, *MBL2*, *SERPING1*, *VSIG4*, *DGKE*, *PLG*, *VWF*, *THBD*, *ADAMTS13* and *VTN.* Genomic DNA was prepared from peripheral blood cells according to standard procedures. Targeted sequences were captured using the Nextera rapid capture custom Enrichment Kit from Illumina and sequencing data generated using a Miseq machine and the Miseq reagent kit v2 (300 cycles). Sequence data were analyzed using the Burrows–Wheeler Alignment and Picard software with additional filtering using custom tools. Variant calling was performed both with bcftools and VarScan and the variant calling files generated merged in one single file using customs tools. Common variants with a minor allele frequency value >1% in any population were excluded. To identify novel and/or pathogenic variants we used different databases: the Exome Aggregation Consortium (ExAC), the Genome Aggregation Database (gnomAD), 1000 Genomes, National Center for Biotechnology Information (NCBI) dbSNP, atypical hemolytic uremic syndrome (aHUS) mutation database (www.fh-hus.org) or our in-house aHUS/C3 glomerulopathy (C3G) database. The patient was also genotyped for the three polymorphisms in the *CR1* gene that allow discrimination of the *CR1-H* and *CR1-L* alleles; the HindIII restriction fragment length polymorphism (RFLP) (intron 27), the H1208R variant (exon 22) and the P1827R variant (exon 33) (10).

### Flow cytometry

Erythrocytes from PNH samples were harvested by centrifugation, washed with phosphate buffered saline (PBS) several times until the supernatant remained clear and stored for up to a week in acid-citrate-dextrose solution A (ACD-A) buffer at 4 °C. Deposition of C3 fragments on the PNH erythrocytes was measured by flow cytometry. Briefly, a 0.4% suspension of erythrocytes was incubated with an in-house mouse monoclonal (mAb) anti-human C3 antibody (mAb SIM27.49.5.68) labeled with Alexa 488 (in-house) at 1 μg/ml in PBS for 30 min at room temperature (RT) and with a mouse monoclonal anti-CD59 labeled with R-Phycoerythrin (Sigma-Aldrich).

### Detection of antibodies against pegcetacoplan

To test for the presence of antibodies against pegcetacoplan, we performed a direct enzyme-linked immunosorbent assay (ELISA), coating the plates with pegcetacoplan at 5 μg/ml for 1 h at 4 °C in PBS, pH 7.4. After blocking with TTBSA (50mM Tris, 150mM NaCl, pH 7.4, 0.2% Tween 20, 1% bovine serum albumin (BSA)) buffer, 1:50 and 1:100 dilutions of the patient plasma were added to the wells and incubated for 1 h at RT. Bound antibodies were detected with a horseradish peroxidase (HRP)-conjugated anti-human immunoglobulins (Igs) (1:1000).

### Binding of C3 and C3b to pegcetacoplan

Binding of C3/C3b proteins to pegcetacoplan was measured using an in-house plate assay. Briefly, 96-well plates were coated with 50 μL of pegcetacoplan at 1 μg/mL in PBS overnight. After blocking the plates with TTBSA buffer for 1 h at RT, 50 μL of the C3/C3b proteins was added at different concentrations (from 50nM to 0,03nM), and the plates were incubated for 1 h at RT. Bound C3/C3b was detected using an in-house mouse monoclonal anti-human C3 (mAb SIM27.49.5.68) followed by a HRP-conjugated goat anti-mouse IgG antibody. After incubation with 3,3′,5,5′-tetramethylbenzidine (TMB) substrate, 0.1 M sulfuric acid was added to stop the reaction. Absorbance was measured at 450 nm. For each C3/C3b mutant protein, we calculated the K_D_ and the relative amount of protein as compared to WT protein needed to provide 50% pegcetacoplan-binding (BD50).

### Functional analyses of the C3 and C3b proteins

Wildtype (wt) and patient C3 were purified from plasma containing 20 mM ethylenediaminetetraacetic acid (EDTA) and 10 mM benzamidine. Supernatant of a 10% (w/v) sodium sulphate precipitation was applied to a lysine-sepharose column. The flow through was collected and applied to a diethylaminoethyl (DEAE)-Sepharose anion exchange column. Protein was fractionated using a NaCl gradient and C3-containing fractions were identified by a sandwich ELISA, using a rabbit polyclonal anti human C3 antibody (in house) as the capture antibody and the mAb SIM27.49.5.68 as the detection antibody, and applied to a Mono S HR 5/5 cation exchange column (GE Healthcare). Protein was eluted and C3-containing peak fractions were pooled and preserved frozen at -70 °C. C3b was generated by digestion with FB and factor D (FD). All C3b preparations were polished by gel filtration on a Superdex 200 Increase 10/300 column (Cytiva). To compare the activation of wt and patient C3 into C3b, purified C3 (400 nM), FB (30 nM) and FD (2 nM) in 20 mM sodium phosphate buffer, pH 7, 100 mM NaCl and 2 mM MgCl_2_ were incubated in a water bath at 37 °C. Aliquots of 20 μL were extracted from the mix at different times, mixed with sodium dodecyl sulfate–polyacrylamide gel electrophoresis (SDS-PAGE) sample buffer (2% SDS, 62.5 mM Tris, 10% glycerol, 0.75% Bromophenol Blue and 40 mg/mL dithiothreitol) to stop the reaction and loaded into a 10% SDS-PAGE gel. The gels were stained using Coomassie brilliant blue R-250 (BioRad) and digitized using a ChemiDoc Imaging System (BioRad). The cleavage of the C3 α-chain normalized to the C3 β-chain was analyzed using the Image Lab Software (BioRad). Similarly, to test differences in the inactivation of wt and patient C3b by FI in the presence of FH, C3b (500 nM), FH (15 nM) and FI (15 nM) were mixed in 10 mM Hepes, pH 7.5, 150 mM NaCl, 0.02% Tween 20. Mixtures were incubated at 37 °C in a water bath and aliquots of 20 μL were collected at different times. The reaction was stopped by dilution in SDS-PAGE sample buffer and samples analyzed in 10% SDS-PAGE. As before, gels were stained with Coomassie brilliant blue R-250 (BioRad) and proteolysis of C3b determined by analyzing the cleavage of the C3 α’-chain normalized to the C3 β-chain.

### Statistical analysis

Binding of the C3/C3b proteins to pegcetacoplan was tested in triplicate in three independent experiments. Three binding curves were generated in each experiment by plotting the amount of C3/C3b protein bound to immobilized pegcetacoplan (measured as absorbance units at 450 nm) versus the total concentration of C3/C3b added to the wells. The K_D_ for each curve was calculated using the curve-fitting software included in the GraphPad Prism statistical package. A t-test was then used to compare the means of the K_D_ values obtained for the control and mutant C3/C3b proteins in each experiment. P values below 0.05 were considered significant. Similar design was used to compare the cleavage of the α′-chain of the wt and mutant C3b by FI and FH, as well as to compare activation of wild-type and mutant C3 proteins by the C3 convertase. In these cases, the intensities of the C3 α′-chain, normalized to the β-chain (α′/β), were quantified at multiple time points, and differences between wild-type and mutant C3/C3b proteins were assessed using t-tests in GraphPad Prism.

### Study approval

The studies herein described received Institutional Review Board (IRB) approval (Comité de Etica de la Investigacion del Principado de Asturias and Comisión de Bioética, Consejo Superior de Investigaciones Científicas). The patient and his relatives provided informed consent for molecular and genetic studies of the complement system.

## Results

### Complement activity and clinical findings

**Figure 1:**
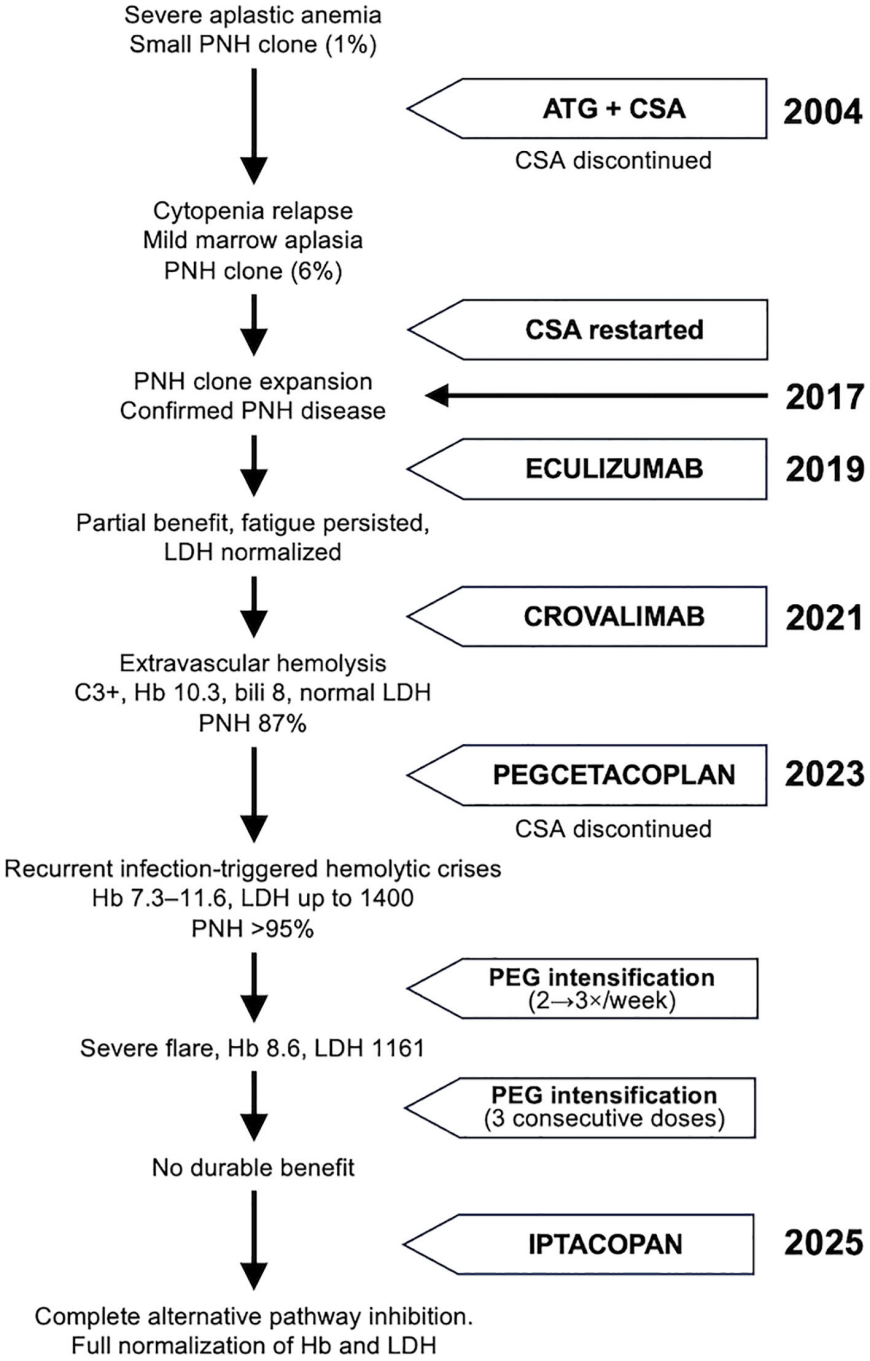
Longitudinal evolution and management of our PNH patient. Figure summarizes the clinical course and the sequential complement inhibitor therapy of our PNH patient from 2004 to 2025 (See Materials and Methods for full details). This figure complements **Table 1** where the hematological parameters and complement activity during pegcetacoplan episodes of intravascular hemolysis are described in detail.

Complement activity was assessed in serial samples from July 2024 to August 2025, during which the patient had multiple intravascular hemolysis episodes (**Table 1**; **Figure 1**), evidenced by Hb drops, reticulocytosis, and elevated LDH. Plasma C3 and properdin levels were consistently high, indicating effective biochemical blockade of C3 activation, with no detectable C3 fragments (iC3b, C3dg) or sC5b-9. Remarkably, AP50 values remained normal throughout, despite increased pegcetacoplan dosing, whereas all other PNH and C3G patients in our laboratory showed undetectable AP50 even on lower pegcetacoplan doses. This preserved AP50 suggests that under strong complement activation, such as infections, pegcetacoplan fails to fully block C3 activation in this patient. We excluded neutralizing anti-pegcetacoplan antibodies as a cause of incomplete response in our patient, since all serum samples tested negative.

**Table 1:** Hematological parameters and complement activity during pegcetacoplan episodes of intravascular hemolysis.

Date	Status	Clone (Neu/Mo)	Bil Ind (0-0.9mg/dL)	Ret (0.5-2%)	Hb (13-18g/dL)	LDH (135-225U/L)	C3 (68-170mg/dL)	FP (5-15µg/mL)	C3dg (<150UA)	sC5b-9 (<180ng/mL)	AP50 (124-84)
Jul 10, 2024	Asymptomatic	>95%	0.7	2.84	11.9	208	393	24	0	0	NT
Oct 14, 2024	Clinical signs of hemolysis	>95%	5.3	3.26	11.7	1242	378	24	0	0	132
Nov 4, 2024	Asymptomatic	>95%	0.9	2.81	12.1	237	404	26	7	0	113
Nov 25, 2024	Asymptomatic	>95%	1.6	2.43	12.4	387	379	26	0	0	131
Nov 27, 2024	Clinical signs of hemolysis	>95%	2.8	2.5	11.4	1165	413	25	26	0	147
Dec 13, 2024	Asymptomatic	>95%	2.1	3.12	10.6	869	411	25	15	0	136
Dec 23, 2024	Clinical signs of hemolysis	>95%	0.9	6.85	8.6	1161	436	27	0	0	134
Dec 27, 2024	Clinical signs of hemolysis	>95%	0.9	6.02	9.5	1108	424	21	0	0	129
Jan 22, 2025	Asymptomatic	>95%	0.7	3.07	11.8	324	417	19	46	0	112
Feb 19, 2025	Clinical signs of hemolysis	>95%	2.1	1.79	12.5	519	349	25	6	0	129
Mar 26, 2025	Asymptomatic	>95%	1.4	1.77	13	204	349	21	15	NT	47
May 5, 2025	Asymptomatic	>95%	1.3	1.79	13.2	222	292	23	20	0	82
Jun 6, 2025	Asymptomatic	>95%	1.4	2.05	12.9	240	294	34	NT	NT	NT
Jun 24, 2025	Clinical signs of hemolysis	>95%	1.5	2.1	12.4	430	321	23	5	130	130
Aug 20, 2025	Clinical signs of hemolysis	>95%	7.8	3.4	10,4	3100	222	18	7	46	136

NT, Not tested; Bil Ind, indirect bilirubin; Ret, reticulocytes; Hb, hemoglobin; LDH, lactate dehydrogenase; C3, complement C3; FP, Properdin; C3dg, C3 activated fragment C3dg; sC5b-9, Soluble C5b-9 complex; AP50, Alternative pathway 50% hemolytic complement activity test.

### Genetic findings

A comprehensive genetic analysis yielded three key findings: a pathogenic *PIGA* mutation (c.776del; p.Phe259Serfs*2), confirming the diagnosis of PNH; heterozygosity for the *CR1* low-expression allele (*CR1-L*), a variant previously associated with increased C3 opsonization and extravascular hemolysis under C5 inhibition (8, 9); and a rare heterozygous variant in *C3* (c.1514G>A; p.R505H), previously linked to aHUS (unpublished data; **Table 2**), which was postulated to contribute to complement dysregulation. The patient inherited this variant from his asymptomatic mother, who is also heterozygous for the C3_R505H_ variant.

**Table 2:** Functional characterization of C3 Mutations.

Mutation	Disease associated	C3 domain	Complement regulation	Pegcetacoplan binding	Ref.
C3_R505H_	aHUS PNH	MG5	Impaired	Impaired	This report
C3_R161W_	aHUS	MG2	Impaired	Impaired	(19)
C3_R592W_	aHUS	MG6	Impaired	Impaired	(19)
C3_H1157T_	aHUS	TED	Impaired	Normal	(19)
C3_delDG923_	aHUS	MG7	Impaired	Normal	(20)

MG, macroglobulin domain; TED, thioester-containing domain.

### Functional characterization of the C3R505H variant

**Figure 2:**
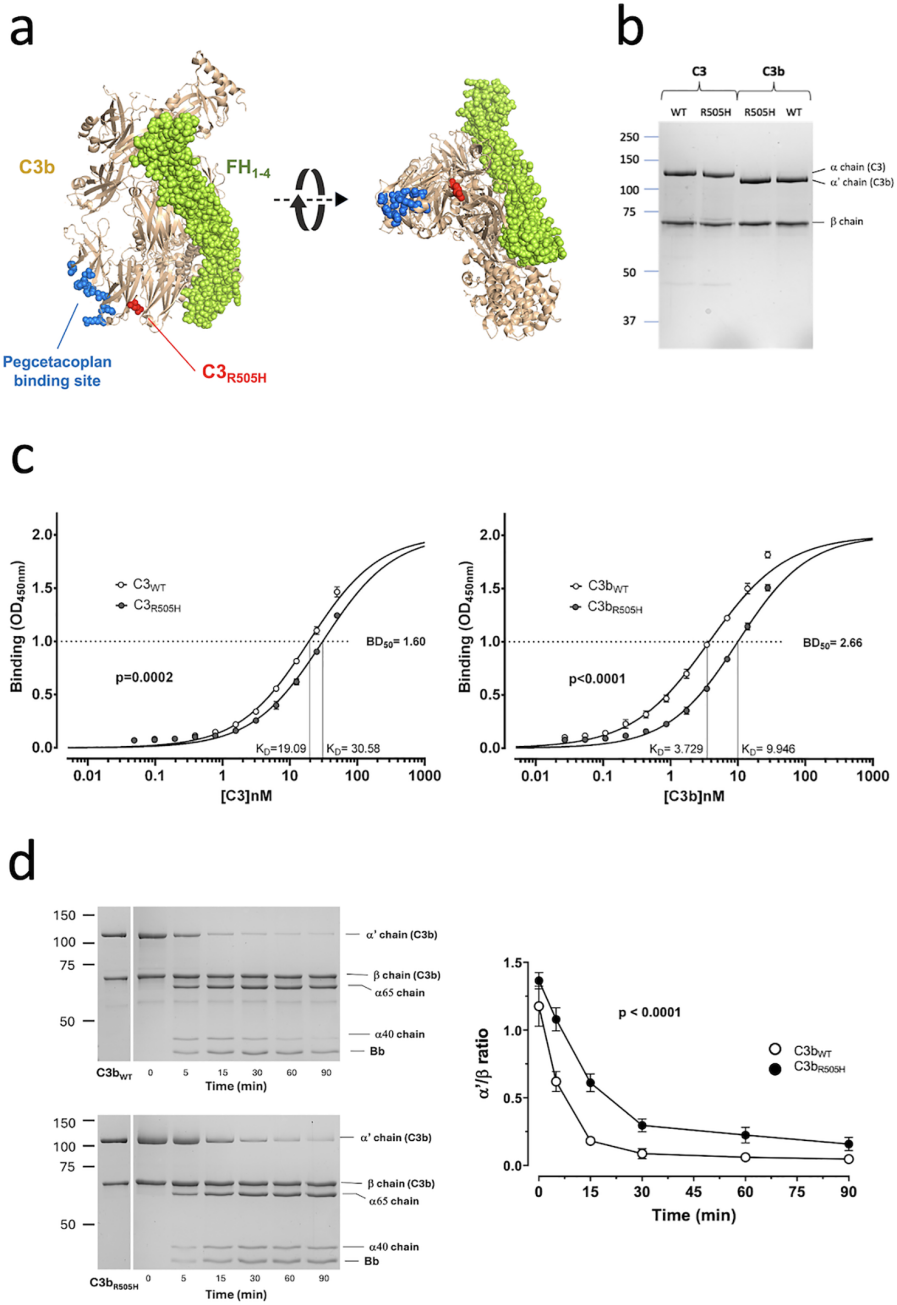
Structural and functional characterization of the C3_R505H_ mutation. **(a)** Location of the mutation in C3b. Representation of the C3b molecule (beige) with the position of the C3_R505H_ variant indicated in red. The N-terminal region (SCR_1-4_) of FH interacting with the C3b molecule is shown in green. The amino acid residues in the C3b molecule relevant for the binding of pegcetacoplan are shown in blue. Structure drawn with Pymol from PDB 2WII. **(b)** Sodium dodecyl sulfate–polyacrylamide gel electrophoresis (SDS–PAGE) analysis of C3 purified from the patient’s mother and a control individual, and the C3b generated from these proteins. C3 presents two bands corresponding to the α chain (~115 kDa) and the β chain (~75 kDa). When C3 is activated by the C3 convertase, the α chain is cleaved to generate the α′ chain (~110 kDa), resulting in the formation of C3b and the release of the C3a (~5 kDa) fragment. **(c)** Binding of C3 (left) and C3b (right) to pegcetacoplan was analyzed in plate assays as described in Materials and Methods. Briefly, increasing amounts of C3 (or C3b) were added to ELISA plates coated with pegcetacoplan, and bound C3 (or C3b) was detected using a mAb against human C3 followed by an HRP-labeled secondary antibody against mouse Igs. Binding curves were obtained by plotting the amount of C3/C3b bound to immobilized pegcetacoplan (measured as absorbance units at 450 nm) versus the total concentration of C3/C3b added to the wells. The apparent dissociation constant (K_D_) for the interaction of each protein with pegcetacoplan was calculated using the curve-fitting tools in the GraphPad Prism statistical software. The rightward shift of the curves obtained for the mutant proteins indicates a lower affinity for pegcetacoplan (i.e., an increased K_D_). This difference is significant for both C3 and C3b proteins. The K_D_ values for all proteins are shown next to the x-axis, along with the K_D_^WT^/K_D_^Mutant^ (BD50) ratios. Experiments were performed in triplicate, and the differences were analyzed by t-test using GraphPad Prism. **(d)** Inactivation of C3b by FI and FH. On SDS–PAGE, C3b displays two bands corresponding to the α′ chain (~110 kDa) and the β chain (~75 kDa). Upon inactivation by Factor I (FI) in the presence of Factor H (FH), the α′ chain is proteolyzed into two main fragments of approximately 65 kDa and 40 kDa, corresponding to the generation of iC3b. On the left, a Coomassie-stained gel illustrates that the α′ chain of C3b generated from the control individual’s C3 is cleaved by FI and FH more rapidly than the α′ chain of C3b derived from the patient’s mother (bottom). This indicates that the mutant C3b protein is inactivated more slowly than the wild-type (WT) C3b protein. On the right, the curves show the disappearance of the α′ chain, normalized to the β chain (α′/β ratio), demonstrating that the difference is statistically significant (p < 0.0001). Experiments were performed in triplicate, and differences were analyzed using an unpaired t-test in GraphPad Prism.

Structural analysis placed the C3_R505H_ variant in the macroglobulin 5 (MG5) domain, within the central MG-ring of C3 (MG1 to MG6 domains), between the pegcetacoplan-binding site and the FH-interaction region, suggesting effects on both (**Figure 2a**). To test this, we purified the mutant and wild-type C3 proteins from the patient’s asymptomatic mother and a normal control individual, respectively, and activated some of the purified proteins to C3b (**Figure 2b**). The mother’s C3 and C3b preparations contained both wild-type and mutant protein, because separation was not possible. Nevertheless, when we evaluated the binding of the C3 proteins to plates coated with pegcetacoplan, her C3 and C3b preparations bound with only about half the affinity of control C3, indicating that the C3_R505H_ variant has impaired pegcetacoplan binding (**Figure 2c**).

We next compared inactivation of mutant and wild-type C3b by FI in the presence of FH. Despite the mixed composition of the mother’s preparation, the data show that C3b_R505H_ is more resistant to FI/FH inactivation than wild-type C3b (**Figure 2d**). Overall, these assays confirm that C3_R505H_ impairs both complement regulation on E-PNH and pegcetacoplan binding, explaining the patient’s excessive extravascular hemolysis under terminal inhibitors and intravascular hemolysis under pegcetacoplan.

### Broader implications of MG-ring variants

**Figure 3:**
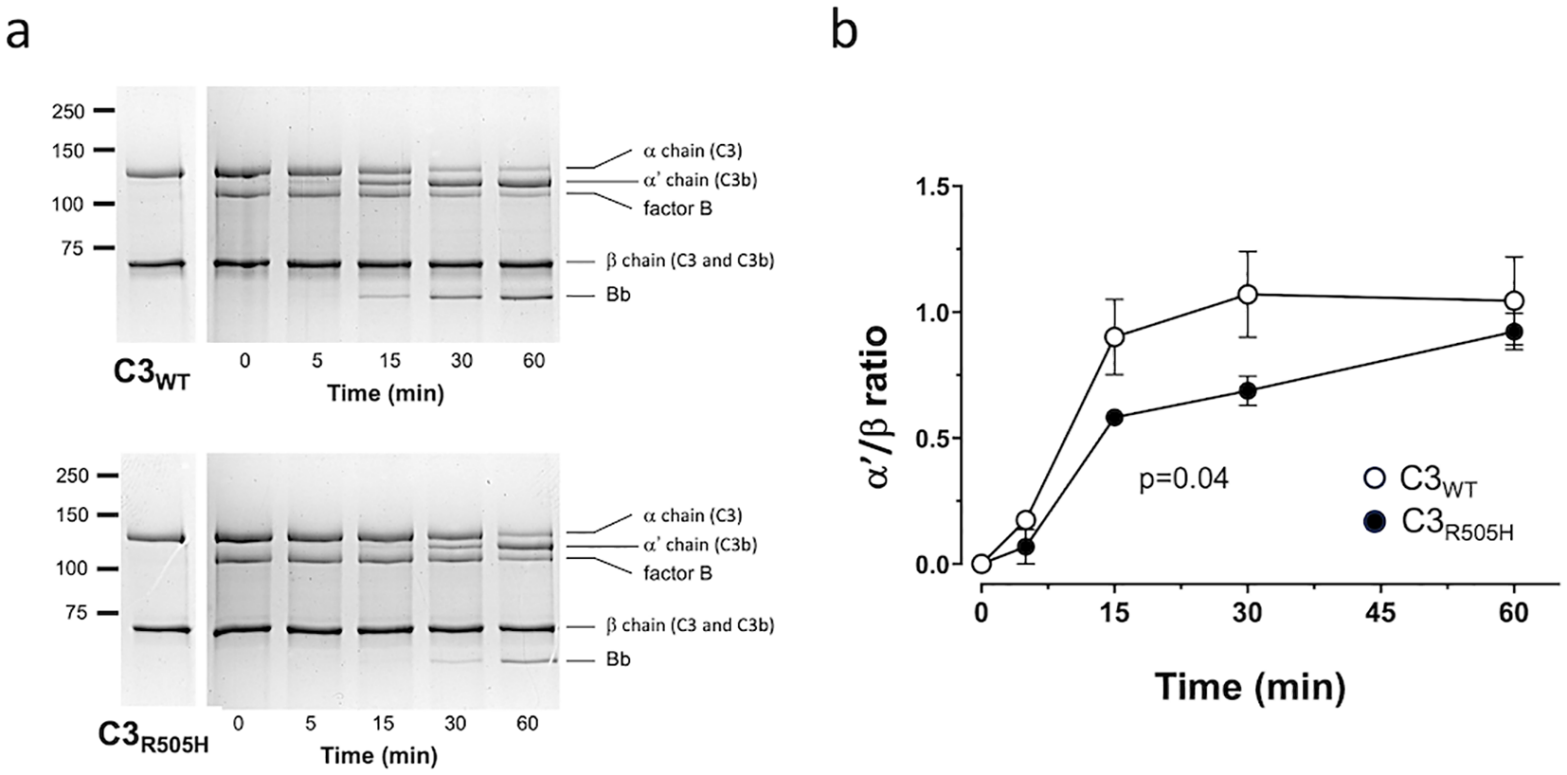
C3_R505H_ is activated more slowly than C3WT by the AP C3-convertase. On SDS–PAGE, C3 presents two bands corresponding to the α chain (~115 kDa) and the β chain (~75 kDa). When C3 is activated by the C3 convertase, the α chain is cleaved to generate the α′ chain, resulting in the formation of C3b and the release of the C3a (~5 kDa) fragment. On the left **(a)**, Coomassie-stained gels show a representative time course experiment illustrating how the α chain of the control individual’s C3 is proteolyzed to the α′ chain more rapidly than the α chain of the C3 generated from the patient’s mother (bottom). On the right **(b)** are curves from a time course experiment showing that the differences in C3b generation, measured as the appearance of the α′ chain, normalized to the β chain (α′/β chain), are significantly different (p=0.04). Experiments were performed in triplicate, and the differences were analyzed by t-test using GraphPad Prism.

**Figure 4:**
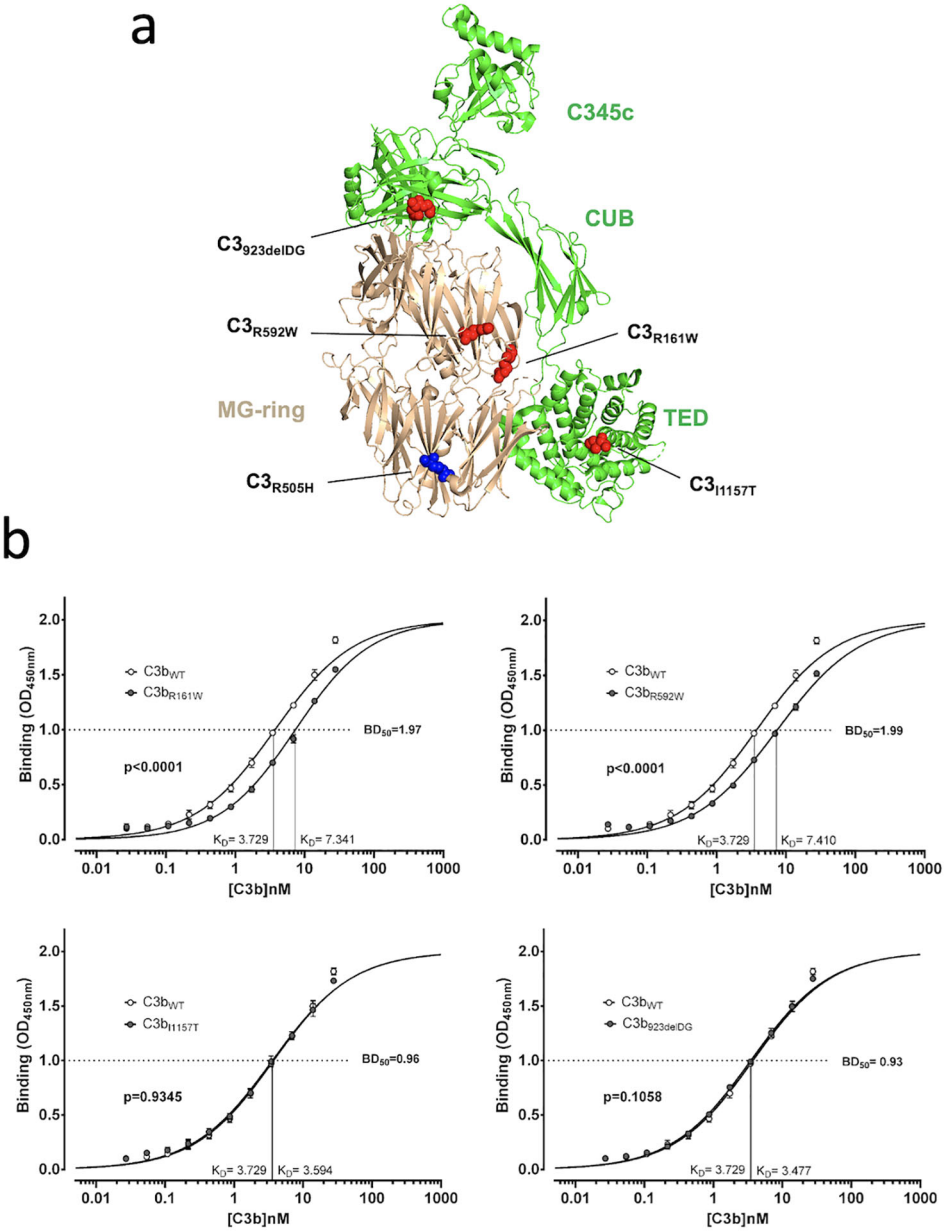
Binding to pegcetacoplan of the C3b proteins generated from different heterozygous C3 GOF mutants. **(a)** Location of the mutations in the C3b protein. Representation of the C3b molecule (green) with the MG-ring in beige, and the C345C, CUB and TED domain indicated. The position of the C3_R505H_ variant is indicated in blue. All other mutations are indicated in red. Structure drawn with Pymol from PDB 5FO7. **(b)** Binding of the four C3b mutants to pegcetacoplan was assessed in plate assays as described in the Materials and Methods section and in the footnote of **Figure 2c**. The binding curves for each C3b mutant (black circles) are shown together with the curve for control C3b (white circles). The rightward shift of the curves for the mutant proteins C3b_R161W_ and C3b_R592W_ indicates a lower affinity (increased K_D_) for pegcetacoplan compared with C3b_WT_. No differences were observed between C3b_WT_ and the mutant proteins C3b_ΔDG923_ and C3b_I1157T_. The K_D_ values for all proteins are indicated next to the x-axis, along with the K_D_^WT^/K_D_^Mutant^ (BD50) ratios. All experiments were performed in triplicate, and differences were analyzed using an unpaired t-test in GraphPad Prism.

The C3_R505H_ variant in MG5, although distant from pegcetacoplan and FH binding surfaces (**Figure 1a**), still significantly impacts both interactions. This suggests that it is sufficient to alter the structure of the C3 MG-ring enough to potentially impair other functions, such as its interaction with the C3 convertase. To test this possibility, we analyzed the activation of C3_WT_ and the C3_R505H_ by the C3 convertase and found that the latter was activated significantly more slowly (**Figure 3**). Then, we hypothesized that other C3 GOF variants in the MG-ring, previously identified in our laboratory and known to affect regulation by FI and the complement regulator membrane cofactor protein (MCP), might also significantly alter the MG-ring with consequences for pegcetacoplan binding. We examined four additional C3 GOF variants: C3_R161W_ and C3_R592W_, located in the MG2 and MG6 domains, respectively, within the MG-ring, and C3_delDG923_ and C3_I1157T_, in the MG7 and the thioester-containing domain (TED) domains, respectively, as negative controls (**Figure 4a**) (**Table 2**). For these experiments, we used plasma-purified proteins generated during earlier functional studies and stored at –80 °C (19–21). Notably, C3_R161W_ and C3_R592W_ displayed reduced pegcetacoplan binding (**Figure 4b**), confirming that other C3 MG-ring GOF mutations can impair this interaction, whereas C3_delDG923_ and C3_I1157T_, which lie outside the MG-ring, bound pegcetacoplan normally. These results support the concept that structural alterations in the C3 MG-ring represent a broader mechanism of resistance to pegcetacoplan.

## Discussion

Incomplete responses to complement inhibition in PNH are increasingly recognized and remain unpredictable. Terminal inhibitors such as eculizumab effectively block C5 cleavage and intravascular hemolysis, yet many patients still develop anemia, reticulocytosis, and fatigue from C3-mediated extravascular hemolysis. Proximal inhibitors like pegcetacoplan, which act upstream at C3 to control both intra- and extravascular hemolysis, also leave a subset of patients with intravascular hemolysis episodes (18), often attributed to pharmacokinetics, though the precise molecular basis remains unclear.

Our study describes the first PNH case in which the cause of incomplete responses to complement inhibition is fully documented at the molecular level. We identified a rare C3 GOF mutation (C3_R505H_) that directly impairs both the physiological regulation of C3b by FI/FH and the pharmacological blockade by pegcetacoplan, explaining the occurrence of both extravascular hemolysis under terminal inhibitors and intravascular hemolysis under pegcetacoplan.

The location of the C3_R505H_ mutation within the central MG-ring of C3 reveals an important and unexpected structural particularity. The MG-ring, organized by six macroglobulin (MG) domains (MG1-MG6), serves as a multifunctional hub of the C3 molecule, participating in the binding of complement regulators, mediating the interaction between the C3 substrate and the C3 convertase, and providing the binding site for pegcetacoplan (22, 23). Because the C3_R505H_ mutation impacts all these functions, we hypothesized that the MG-ring is particularly sensitive to perturbations within individual MG domains. This is especially relevant for pegcetacoplan, as its binding site lies in the groove between the MG4 and MG5 domains (23), a region that may be particularly vulnerable to subtle conformational changes. Additionally, pegcetacoplan binding affinity for C3 and C3b is not extraordinarily high (nM) (24), which may render it more susceptible to these subtle structural perturbations. Supporting this, we show that additional C3 GOF variants, such as C3_R161W_ and C3_R592W_, previously shown in our laboratory to interfere with the binding of the MCP complement regulator (19), also display impaired pegcetacoplan binding, something that does not occur for C3 GOF variants like C3_I1157T_ and C3_delDG923_ outside the MG-ring. Notably, the three C3 variants impacting the pegcetacoplan binding described here (C3_R161W_, C3_R505H,_ and C3_R592W_) involve substitution of arginine residues, which are typically engaged in salt bridges that stabilize domain interfaces and maintain allosteric pathways and long-range communication (25, 26). These observations underscore that mutations across different MG domains of the MG-ring can weaken pegcetacoplan binding and highlight the structural vulnerability of the MG-ring as a whole.

Our findings therefore demonstrate that C3_R505H_ is not an isolated anecdote. Notably, analysis of the gnomAD v4.1.0 dataset shows that roughly 1 in 125 individuals (≈0.8%) in the general population carries a rare (<0.1%) nonsynonymous C3 variant affecting a residue within MG1–MG6, underscoring the potential relevance of this resistance mechanism. Moreover, we and others demonstrate that C3 gain-of-function mutations located within the MG-ring domains (such as C3_R161W_, C3_R505H_, and C3_R592W_) that impair complement regulation show increased prevalence in pathologies precisely susceptible to treatment with pegcetacoplan.

Taken together, these observations highlight the importance of comprehensive complement and genetic analyses in patients with suboptimal responses to complement inhibition. Personalized treatment strategies informed by molecular testing will likely become increasingly relevant as therapeutic blockage of C3 in PNH and other complement-mediated pathologies expands. In this context, functional readouts such as the AP50 assay may provide a valuable biomarker to monitor the efficacy of pegcetacoplan and detect residual complement activity that could drive breakthrough hemolysis.

It is important to note that the clinical consequences of C3 MG-ring variants may differ substantially depending on the disease context. In PNH patients treated with pegcetacoplan, virtually all erythrocytes are GPI-deficient and thus lack CD55 and CD59 protection, rendering them extremely vulnerable to complement activation. In this setting, heterozygous C3 variants that impair pegcetacoplan binding could make the PNH patient exceptionally susceptible to intravascular hemolysis crises, particularly during acute-phase responses or infections that transiently increase C3 synthesis. In contrast, in diseases such as aHUS or C3G, where complement activation primarily occurs on endothelial cell surfaces or in fluid phase, the same variants may have distinct or less pronounced therapeutic implications. Therefore, while our findings provide a mechanistic framework for understanding incomplete responses in PNH, they also underscore the need to investigate the functional and clinical impact of C3 MG-ring variants in other complement-mediated diseases.

In summary, this work not only explains an individual patient’s incomplete responses but also highlights a broader principle: genetic variation in complement proteins can profoundly shape therapeutic outcomes in complement-mediated diseases. It also uncovers previously unrecognized structural aspects of the C3 molecule, revealing the MG-ring’s structural sensitivity to genetic variants, with important consequences for pegcetacoplan binding. These insights underscore the need to incorporate genetic and functional analyses into future evaluations to optimize personalized anti-complement therapies. While our study provides robust functional evidence linking C3 MG-ring variants to impaired complement regulation and reduced pegcetacoplan binding, clinical correlations were limited to the index PNH case. Therefore, broader clinical validation in larger patient cohorts and in other complement-mediated diseases will be necessary to fully establish the translational relevance of these findings.

## Data Availability

The raw data supporting the conclusions of this article will be made available by the authors, without undue reservation.
